# HPV infection in oral cancer, our experience: prevalence, clinical implications, and current vaccination program in Spain

**DOI:** 10.4317/jced.60514

**Published:** 2023-07-01

**Authors:** Íñigo Aragón-Niño, Carolina Cuesta-Urquía, Javier González-Martín-Moro, María-José Morán-Soto, José-Juan Pozo-Kreilinger, Marta-María Pampín-Martinez, José-Luis del Castillo-Pardo-de Vera, José-Luis Cebrián-Carretero

**Affiliations:** 1 Medical Resident. Oral and Maxillofacial Surgery Department. La Paz University Hospital. Madrid, Spain; 2 Physician attending / Faculty. Oral and Maxillofacial Surgery Department. La Paz University Hospital. Madrid, Spain; 3 Physician attending / Faculty. Pathology Department. La Paz University Hospital. Madrid, Spain; 4 Head of the Department. Oral and Maxillofacial Surgery Department. La Paz University Hospital. Madrid, Spain

## Abstract

**Background:**

Oral cancer is the 11th most common type of cancer in the world, with established major risk factors as tobacco and alcohol, and recently included high-risk human papillomavirus types 16 and 18. HPV types 16 and 18 are the etiologic agents of cervical cancers and a proportion of oropharyngeal cancers. However, the picture of HPV and the clinical implications of oral cancers are not clear with most reports combining oral cancer data with head and neck cancers. It has been confirmed as a favorable prognostic factor in oropharyngeal cancer. However, the prognostic value of HPV in oral squamous cell carcinoma is still unclear.

**Material and Methods:**

The main objective of this article is to present the evidence encountered following a bibliographical review of recent publications specifically related to oral cancer and its differences from oropharyngeal cancer. The secondary goals are to present the findings of a five-year retrospective observational study of the prevalence of HPV infection in oral cancer patients treated by the Oral and Maxillofacial Surgery Department at La Paz University Hospital (Madrid, Spain), and finally, we to evaluate and compare our country’s HPV prevention program in comparison to other European countries.

**Results:**

According to the review of the literature, HPV positive oral squamous cell carcinoma is associated with significantly decreased overall survival and distant control. Bibliographic review suggest HPV infection can be used as a negative prognostic factor in oral squamous cell carcinoma.

**Conclusions:**

As regards diagnostic testing for HPV, it should be extended to as many cases of oral cavity squamous cell carcinoma as possible, especially in those with risk factors. The current vaccination program in Spain does not have adequate coverage and is significantly under the level of other European Union countries; it should be expanded and catch-up strategies should be included.

** Key words:**HPV, OSSC, Papillomavirus, oral carcinoma, prevention.

## Introduction

The first bibliographic reference associating HPV infection and oral cancer is from 1983 by Dr. Syrjanen (Finland) (1), who studied 40 cases of epidermoid carcinoma in the oral cavity and found histologic features suggestive of HPV infection in up to 22.5% of the samples analyzed.

Currently, oral cancer is the 11th most frequent cancer in the world and its most important associated risk factors are tobacco and alcohol. However, in recent years, infection by high-risk types of human papillomavirus, especially 16 and 18, has been gaining importance. This risk factor has even surpassed toxic habits in countries such as the United States (2,3).

There is sufficient published literature to demonstrate that HPV types 16 and 18 infection is an etiologic agent of cervical and oropharyngeal cancer (4-10). The mechanism for the induction of carcinogenesis lies in the role of the E6 and E7 proteins (11-14).

Specifically, the E6 protein degrades the tumor suppressor p53, inhibits cellular proapoptotic proteins and activates telomerase, which is related to cellular immortality.

In addition, the E7 protein inactivates the retinoblastoma protein, which has a suppressor role in tumor activity. Under normal conditions p16 binds to the cyclin D1 complex to prevent phosphorylation of the retinoblastoma protein; for this reason inhibition of the RET protein by HPV infection produces a measurable increase in p16 and is used as a diagnostic test (15-17).

Furthermore, in the same way, the E7 protein increases the activity of the myc gene with the effect of increasing cellular activity.

All published literature agrees on a direct relationship between the risk of cervicofacial cancer and HPV infection (9). However, it is difficult to find isolated data on the relationship between infection and oral cancer and its implications since most studies combine data on oral cavity cancers (tongue, jugal mucosa, retromolar trigone, floor of the mouth and lip) with data on other head and neck cancers (pharynx and oropharynx, including soft palate).

Moreover, according to the available results HPV infection is considered as a positive prognostic factor in some head and neck cancers such as oropharyngeal cancer(18). However, the prognostic value in relation, in isolation, to oral cancer is still unclear (19-23).

The main objective of this article is to present the evidence found following a bibliographical review of recent publications specifically related to oral cancer and its differences from oropharyngeal cancer.

The secondary goals are to present the findings of a five-year retrospective observational study of the prevalence of HPV infection in oral cancer patients treated by the Oral and Maxillofacial Surgery Department at La Paz University Hospital (Madrid, Spain), and finally, we to evaluate and compare our country’s HPV prevention program in comparison to other European countries.

## Material and Methods

For the literature review we used the PubMed search engine for articles indexed in the US National Library of Medicine, National Institute of Health, NIH/NLM, USA. An advanced search was performed with the following query: ((((human papilloma virus) OR (vph)) AND (oral cancer)) NOT (oropharyngeal)). A total of 837 results were obtained.

The search was then restricted to articles published between 2016 and 2022 and 339 articles were obtained. Of these, 47 were selected for their relevance, demographic criteria, and applicability to the objective of this review.

**Figure 1:**
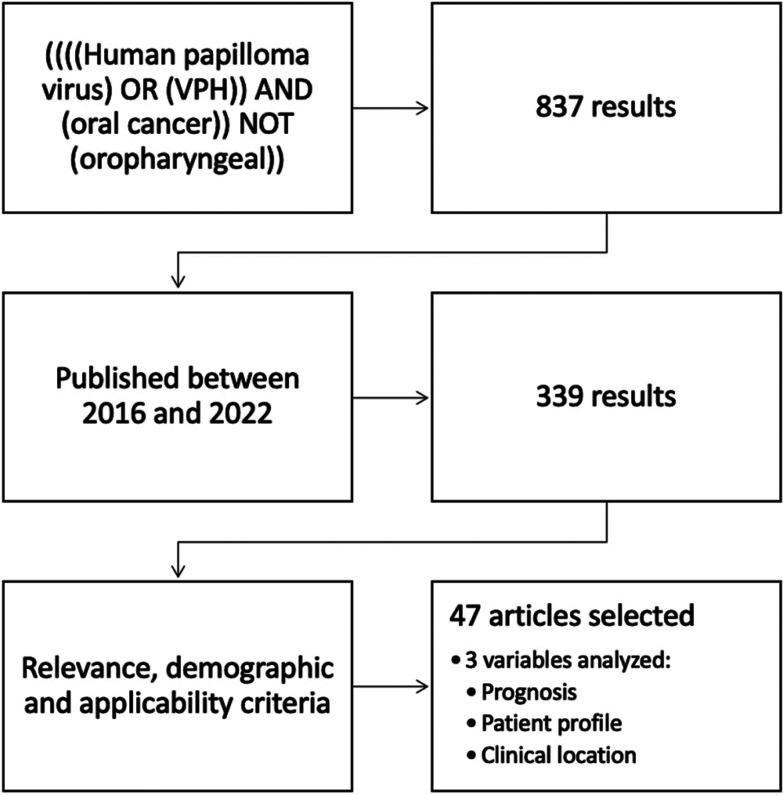
Article selection diagram.

These articles were analyzed to find differences between HPV-positive oral cavity epidermoid carcinoma cases and HPV-negative cases in terms of three variables: patient profile, clinical location, and prognosis, (Fig. 1).

This review is complemented by data from our own clinical experience in the form of a retrospective descriptive study of all patients treated by the Oral and Maxillofacial Surgery Service of La Paz University Hospital, between February 2017 and February 2022 determining how many cases were tested for HPV and the results. A total of 429 samples and a total of 135 p16 tests performed were included.

Finally, the guidelines published by the interterritorial health council of Spain and by the different health agencies of the European Union countries were reviewed to analyze and compare the different prevention plans against HPV infection between Spain and the other European Union countries.

## Results

The results obtained from the literature review are:

- Patient profile(18,24):

o It stands out that patients with epidermoid carcinoma of the oral cavity with HPV infection were less related to toxic habits of tobacco consumption (42.1% vs. 59.6%) and alcohol (5.3% vs. 22.1%).

o Predominance of male sex (73.7% vs. 68.4%).

o Lower age (61.9 +- 12.3 vs. 62.8 +-13.6).

- Location(21,24,25):

o There is an increased risk of oral cancer in the tongue, palate and floor of the mouth.

o Specifically and separately, E7 protein positivity for HPV serotype 16 implies an OR 2.71 for oral floor cancer and OR 3.34 for oral palate cancer.

- Prognostic(26–29):

o A systematic review of 2126 studies determines that the relationship between HPV infection and oral cavity squamous cell carcinoma implies a lower overall survival (OS) HR= 1.45, (95% CI 1.10-1.93) and lower distant disease control (DC) HR = 2.16, 95% (CI, 1.54-3.04).

o Furthermore HPV-16 is a worse predictor at 5 years for:

• Overall survival in uncomplicated disease (*p*=0.075).

• Overall survival in advanced disease (*p*=0.113).

• Early metastatic disease.

In the descriptive retrospective study carried out in our service, a total of 429 histological samples from patients who were intervened between February 2017 and February 2022 were included. Of the total number of samples included, the p16 diagnostic test was performed in 135 samples, representing 31%.

**Figure 2:**
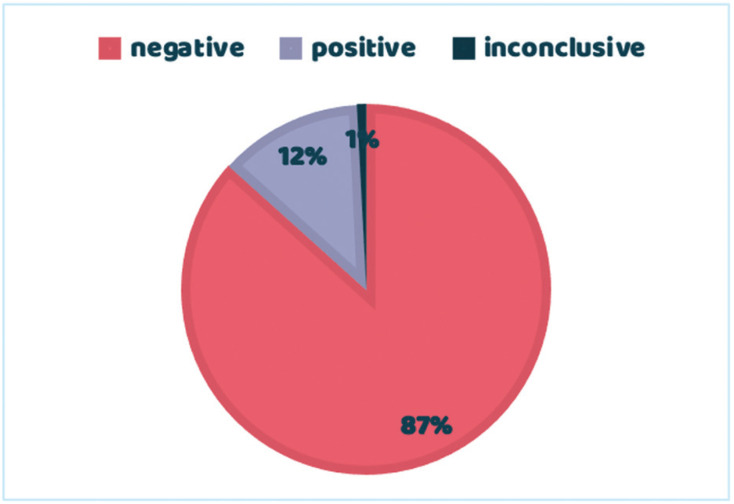
Overall results of samples tested with p16.

Of the total number of samples in which p16 testing was performed, a positive result was obtained in 17, negative in 117 and inconclusive in 1, (Table 1, Fig. 2).

**Table 1:**
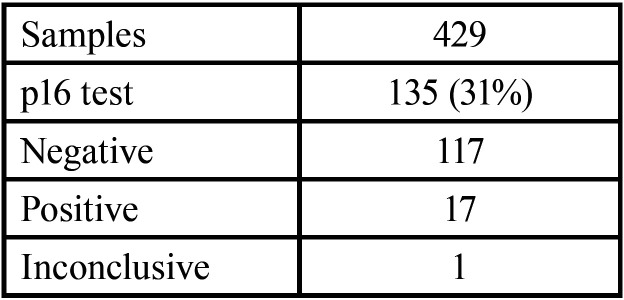
Overall results of the samples included in the study.

Analyzing each year independently, in 2017 (from February) 65 samples were sent, the test was performed in 31 (47%), with negative result in 29 and positive in 2. In 2018, 70 samples were sent, the test was performed in 20 (28%), with negative result in all. In 2019, 131 samples were sent, 32 (24%) were tested, with negative results in 27 and positive in 5. In 2020, 68 samples were sent, 20 (29%) were tested, with negative results in 18 and positive in 2. It should be pointed out that there was a decrease in surgical and healthcare activity during this period due to the COVID-19 pandemic.

In 2021, 84 samples were sent, 30 (35%) were tested, with negative results in 21, positive in 8 and inconclusive in 1. In 2022 (up to February), 11 samples were sent, 1 was tested with a negative result, (Table 2).

**Table 2:**
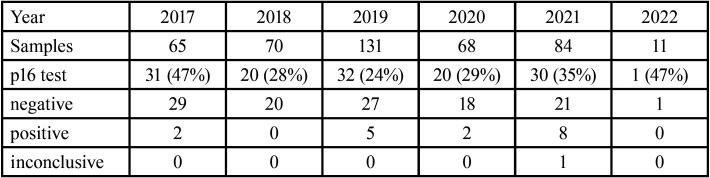
Annual distribution of results.

**Figure 3:**
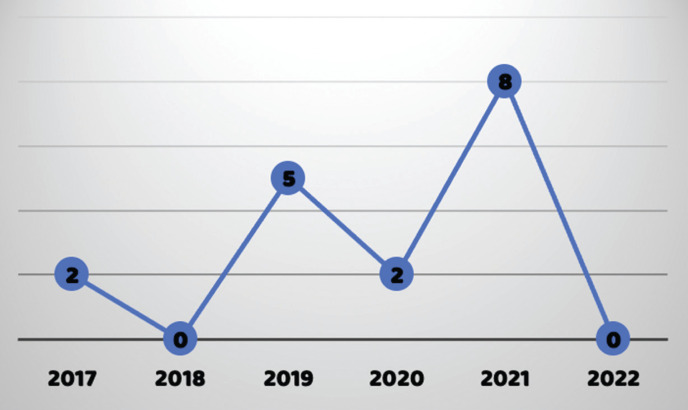
Positive results by year of collection (from February 2017 to February 2022).

Analyzing the positive results individually, the year with the most positive results was 2021 and the one with the least was 2018, (Table 3, Fig. 3).

**Table 3:**
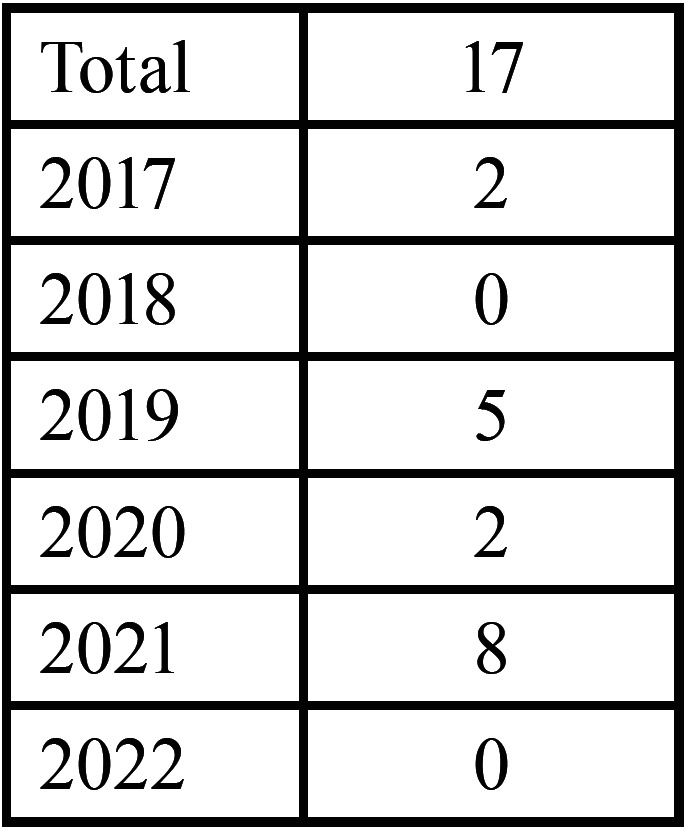
Positive results per year.

The vaccination schedule published by the interterritorial council of the Spanish national health system includes vaccination against HPV at 12 years of age for girls only, with two doses separated by at least 5-6 months.

After 12 years of age and up to 18 years of age, it includes vaccination only for women who have not been vaccinated, or have been partially vaccinated, previously.

As for adult vaccination (from 18 years of age), it is only contemplated in the population at risk, both men and women who meet at least one of the following conditions:

- WHIM syndrome (IDP): vaccine covering types 6 and 11.

- HIV infection, up to 26 years of age.

- Men who have sex with men, up to 26 years of age.

- Persons in prostitution, up to 26 years of age.

- Women with cervical excisional treatment (any age).

If we review the vaccination program of other European countries, most of them include vaccination between 10 and 14 years of age for males and females. Many also have catch-up strategies to rescue those adults not vaccinated during childhood.

According to the available literature there is high evidence that vaccination is an effective tool in protecting against the development of malignant and premalignant lesions, being especially useful in reducing transmission (20,30,31).

Available cost-effectiveness studies indicate that increasing vaccination coverage is cost-effective, especially in the male population (30,32).

## Discussion

There is a paradigm change in the available evidence on risk factors specifically for oral cavity cancer. While HPV infection is widely accepted as a positive prognostic factor in the prognosis of cervicofacial cancer globally, in particular in oral cavity cancer, specifically, it seems to be related to more disseminated disease and a lower overall survival, and can therefore be considered as a negative prognostic factor.

This specific characteristic of oral cancer, which makes it different within the group of cervicofacial cancer, makes it advisable to conduct more studies to analyze in depth this discrepancy and its consequences.

One of the first needs is to have well collected the prevalence of HPV in all possible cases. This makes it especially profitable to perform specific p16 tests on histological specimens from oral cancer resection surgeries and also on pre-surgical diagnostic biopsies.

Until now the usual protocol contemplates the performance of p16 testing only in cases of high suspicion, but analyzing the data of our study we see that in the years that more tests have been performed there are more positive results.

Taking into account that it is a rapid test with an affordable cost and its proven specific prognostic factor in oral cancer, it seems to be a recommendation to extend the protocol and perform this test to all the histological samples studied.

As regards prevention, the unfavorable situation in Spain is notable, especially the absence of childhood vaccination in men, the absence of a “catch-up” program and the limited vaccination coverage in adults, non-existent outside cases of risk.

With the proven efficacy of vaccination against malignant and premalignant disease, and especially against transmission, as well as being a cost-effective strategy, it seems advisable to urgently review the national vaccination program to expand its coverage.
